# Role of C-reactive protein and fibreoptic endoscopic evaluation of swallowing as early markers of stroke-associated pneumonia

**DOI:** 10.3389/fstro.2026.1742758

**Published:** 2026-02-17

**Authors:** Svetlana Politz Geleva, Ludwig D. Schelosky

**Affiliations:** 1 Klinik für Neurologie, Cantonal Hospital of Münsterlingen, Münsterlingen, Switzerland; 2 Klinische Neurologie, District Hospital Rohrbach, Rohrbach-Berg, Austria

**Keywords:** aspiration pneumonia, dysphagia, fees, post-stroke infection, prevention, speech language therapy, stroke, stroke-associated pneumonia

## Abstract

**Introduction:**

Stroke may result in dysphagia, which can subsequently lead to stroke-associated pneumonia (SaP). This condition has been shown to exert a significant negative impact on patient outcome. Early diagnosis and prevention are therefore desirable.

**Methods:**

This retrospective study compared inflammatory markers during the first 4 days after stroke in 515 patients from 2015 and 2021, analyzing associations with dysphagia, year of treatment, dietary adjustments, stroke-associated pneumonia, and antibiotic use. Data entry and descriptive analyses were performed using Microsoft Excel^®^. Datasets from 2015 and 2021 were analyzed in SPSS^®^ (IBM SPSS Statistics 27).

**Results:**

This retrospective analysis demonstrates that dysphagia significantly influences C-reactive protein (CRP) levels within the first 4 days after stroke. Dysphagia and elevated CRP are early markers of emergence of stroke-associated pneumonia, whereas leukocyte count and temperature show limited forewarning value. The combination of post-stroke inflammatory response, dysphagia (PAS > 5), and elevated CRP may serve as an early indicator of SaP and support timely FEES-based assessment.

**Discussion:**

Early elevated CRP levels in dysphagic stroke patients are indicative of aspiration-related inflammation and may serve as a sensitive early biomarker for stroke-associated pneumonia. The combined assessment of dysphagia severity and CRP supports improved early risk stratification and preventive management.

## 1 Introduction

Post-stroke inflammations, and especially SaP, are among the most prevalent causes of mortality subsequent to a cerebrovascular accident (Liu et al., 2018). The acute phase of the disease carries the highest risk of developing SaP. Depending on the amount of aspirated substances, up to 70% of those affected by SaP die (Hannawi et al., 2013).

The acute cerebral lesion has the potential to disrupt the control of defense mechanisms (e.g., reflex closure of the glottis and cough reflex). The resultant dysphagia leads to the aspiration of food, oropharyngeal and gastric secretions, and is a significant risk factor for the development of SaP (Faura et al., 2021). Research indicates that between 19% and 81% of stroke survivors experience significant dysphagia (Saposnik et al., 2008). The development of stroke-induced immunosuppression (SIIS; Vermeij et al., 2013) has been shown to further promote the development of SaP. Elevated CRP levels may indicate infection; however, the role of CRP in cerebrovascular disease remains unclear. The heterogeneous pathophysiology of stroke is reflected in distinct patterns of systemic inflammatory response, which may result from the brain injury itself or from reactions to acute complications and infections (Westendorp et al., 2011; Kalra et al., 2019). The incidence of pneumonia in stroke units ranges from 13% to 33%, with aspirated anaerobes accounting for a significant proportion of the pathogenic germs (Tuttolomondo et al., 2012; Braun et al., 2019).

SaP, in turn, has a significant impact on stroke outcome and, independently of other variables, is linked to almost one third of all deaths following stroke. The pathology and microbiology of stroke-associated pneumonia (SaP) within the first seven days post-stroke remain poorly understood. Smith et al. highlighted the need for improved diagnostic approaches, as chest X-rays show typical findings in only 36% of cases, and propose new imaging-independent SaP criteria (Westendorp et al., 2011; Smith et al., 2015). Elevated CRP levels have been shown to be an indicator of infection (Abele-Horn and Pitten, 2015). Dysphagia was detected in 37–45% of patients using clinical screening, 51–55% with clinical examination, and 64–78% with instrumental diagnostics. Patients with dysphagia had an increased risk of stroke-associated pneumonia (SaP), which was even higher in those who exhibited aspiration during the acute phase of stroke (Martino et al., 2005).

Despite the high incidence and mortality of SaP, there is an absence of a dysphagia management strategy that is evidence-based and integrates early inflammatory markers, such as CRP, with instrumental swallowing assessment using FEES. Current SaP prediction models are predominantly driven by clinical risk factors and do not incorporate objective markers of early pulmonary inflammation or direct visualization of swallowing safety. Consequently, dietary choices—including the utilization or non-utilization of thickened liquids—exhibit significant variability across different stroke units, reflecting a substantial knowledge gap that carries potential implications for patient safety.

The present study has been conducted with the aim of examining the predictive power of CRP in combination with the use of fibreoptic endoscopic evaluation of swallowing (FEES) for the development of stroke-associated pneumonia (SaP) in the acute phase following a stroke.

## 2 Methods

The present study investigated the correlation between the progression of inflammatory parameters (CRP, leukocyte count) and the rise in body temperature in the first 4 days after stroke, and dysphagia and the occurrence of SaP. The diagnostic criteria for aspiration pneumonia remained unchanged in 2015 and 2021. This study aims to evaluate whether adequate dysphagia management reduces the relative risk of aspiration pneumonia and mortality. A secondary objective was to evaluate whether the implementation of routine FEES in the stroke unit in 2021 resulted in a significantly reduced incidence of aspiration pneumonia compared to 2015, when FEES was not utilized. The FEES examination was performed routinely in all stroke patients who exhibited clinical signs of dysphagia during speech-language assessment. Additionally, the study examines whether early post-stroke inflammatory markers (CRP, leukocyte count, temperature) correlate with dysphagia and SaP, and whether infection rates and antibiotic use differed between 2015 and 2021.

Findings are intended to improve patient safety, enhance care quality, and optimize healthcare resource utilization. The study also sought to ascertain whether the number of infections and the therapeutic administration of antibiotics would differ between 2015 and 2021 in the Stroke Unit of the Neurological Clinic of the Cantonal Hospital Münsterlingen.

Laboratory tests were performed on admission (day 1). Subsequent to this, follow-up parameters were taken on days 2–4, depending on clinical necessity, though not routinely. Temperature was measured at least once daily. Following the day of admission blood samples and temperature were taken and measured on a daily basis in the morning, and additionally as clinically indicated; however, no standardized measurement protocol was in place. According to the reference value of the laboratory of the Cantonal Hospital Münsterlingen, CRP is considered normal up to 5 mg/l, and the normal reference range for leukocytes is given as 4–10.5 (10^*^9/l). Body temperature is considered elevated at 37 °C or above.

The data were entered and descriptively analyzed using Microsoft Excel^®^. For the purpose of evaluating the data sets from 2015 and 2021, the data were transferred to SPSS^®^ (Statistical Package for Social Sciences, IBM, SPSS, Statistics 27). The significance level is set at a probability value (*p*-value) of 0.05 or less.

### 2.1 Inclusion criteria

- All patients of the Stroke Unit of the Neurological Clinic of the Cantonal Hospital Münsterlingen in 2015 and 2021

In retrospect, the following variables were extracted from the Swiss Stroke Registry (SSR) for the years 2015 and 2021 (*N* = 515):

- Anonymised SSR identification number
- Sex
- Age
- Details of the stroke (ischaemic, haemorrhagic, etc., anterior or posterior circulation)
- National Institutes of Health Stroke Scale (NIHSS) on admission, discharge and after 90 days
- NIHSS sub-item dysarthria
- Modified Rankin Scale (mRS) prior to the event, on admission, discharge and after 90 days
- Interval between stroke and hospital admission (do patients already arrive at the hospital with SaP due to, for example, a long period of bed rest at home or delayed diagnosis?)
- FEES examination yes/no
- Dysphagia yes/no (Gugging Swallowing Screen (GUSS)/clinical speech therapy assessment/FEES)
- Therapeutic recommendation: nutrition, diet, nasogastric tube (NGS), Percutaneous endoscopic gastrostomy (PEG) tube during hospitalization
- Pneumonia yes/no (definition of pneumonia: diagnosis in discharge letter, fever/CRP/increase in infection parameters without other explanation, use of antibiotics)
- CRP, total leukocyte count and temperature on days 0, 1, 2, 3, 4 after stroke
- Antibiotic therapy (yes/no)
- Decision on discontinuation of therapy (palliative treatment)

The study was registered and approved on the 23rd of February 2023 by EKOS (Ethics Commission of Eastern Switzerland) with BASEC No. Req-2023-00228 EKOS 23/029.

## 3 Results

The study included 515 stroke patients (mean age 73.4 ± 13.0 years; 55.3% male). A total of 467 patients experienced an ischaemic event, defined as amaurosis fugax, transient ischemic attack (TIA) or ischaemic infarction. 40 suffered an intracerebral hemorrhage and 7 had subarachnoid hemorrhage (see Table 1).

**Table 1:** Patient characteristics.

**Characteristic**	**Year**				
		**2015**		**2021**	
		***N***	**%**	***n***	**%**
Sex	Male	137	54.8%	148	55.8%
	Female	113	45.2%	117	44.2%
	Total	250	100.0%	265	100.0%
Age (years)	< 60	43	17.3%	42	15.8%
	60–80	116	46.6%	132	49.8%
	>80	90	36.1%	91	34.3%
	Total	249	100.0%	265	100.0%
Stroke type	Amaurosis fugax	3	1.2%	4	1.5%
	Cerebral venous thrombosis	0	0.0%	1	0.4%
	Hemorrhagic stroke	11	4.4%	29	10.9%
	Ischaemic stroke	186	74.4%	167	63.0%
	Subarachnoid hemorrhage	6	2.4%	1	0.4%
	Transient ischemic attack	44	17.6%	63	23.8%

The severity of neurological deficits, measured using the NIHSS, remained similar in both 2015 and 2021, while mRS data were more severe in 2021 (discharge: 242/256 vs. 197/242; 90 days: 249 vs. 130; see Table 2).

**Table 2:** NIHSS, mRS, CRP, leukocytes and temperature categorized by year.

**Variable**	**Year**						***p*-value**
	**2015**			**2021**			
	**Mean**	**SD**	***n***	**Mean**	**SD**	***n***	
NIHSS upon admission	4.5	6.3	242	4.6	6.0	256	*P* = 0.570
NIHSS before admission	0.3	2.0	221	1.0	3.4	239	*P* = 0.308
NIHSS upon discharge	1.8	3.5	219	1.9	3.7	239	*P* = 0.501
NIHSS after 90 days	0.8	2.5	123	1.1	2.6	207	*P* = 0.038
mRs upon admission	1.3	1.7	202	2.1	1.8	252	*P* < 0.001
mRs before admission	0.5	1.0	205	0.7	1.2	245	*P* = 0.055
mRs upon discharge	1.0	1.5	197	1.7	1.7	242	*P* < 0.001
mRs after 90 days	0.7	1.3	130	1.6	1.9	249	*P* < 0.001
CRP day 1	13.2	29.1	225	16.5	39.7	241	*P* = 0.607
CRP day 2	16.1	29.0	153	28.9	53.8	107	*P* = 0.118
CRP day 3	33.4	55.5	82	38.6	58.9	69	*P* = 0.701
CRP day 4	40.6	49.0	52	47.2	60.9	67	*P* = 0.921
Leukocytes day 1	8.9	3.0	229	8.5	3.2	243	*P* = 0.026
Leukocytes day 2	8.4	3.5	159	8.0	3.1	113	*P* = 0.263
Leukocytes day 3	8.6	3.5	81	8.1	2.7	80	*P* = 0.699
Leukocytes day 4	8.7	3.3	57	8.7	3.2	74	*P* = 0.851
Temperature day 1	36.3	0.6	228	36.5	0.6	239	*P* < 0.001
Temperature day 2	36.4	0.5	218	36.7	0.6	203	*P* < 0.001
Temperature day 3	36.4	0.6	214	36.7	0.5	180	*P* < 0.001
Temperature day 4	36.4	0.6	200	36.7	0.7	169	*P* < 0.001

NIHSS, National Institutes of health stroke scale; mRS, modified rankin scale; CRP, C-related peptide.

CRP, leukocytes, and temperature were measured on admission and on days 2–4 as indicated; only temperature differed significantly between years (see Table 2).

**Figure 1:**
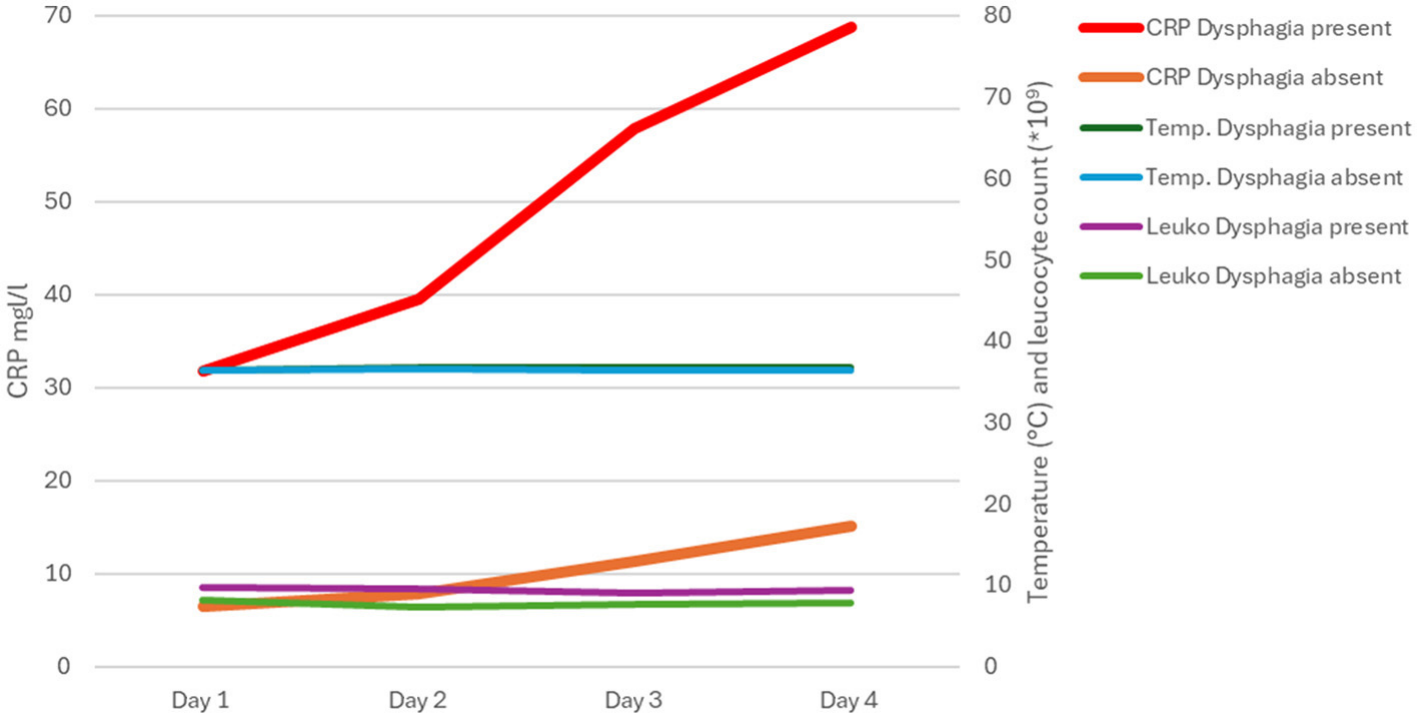
In cases of dysphagia, the CRP value rises rapidly within the initial four days. Leukocytes and temperature do not change significantly.

In patients diagnosed with dysphagia, there is a rapid increase in the CRP value within the initial four days (see Figure 1). The leukocyte count and temperature remained stable over the initial 4 days in both 2015 and 2021 (see Figure 1). Patients without dysphagia exhibited low, but still abnormal, CRP levels in both years (see Figure 1). As shown by logistic regression, patients with dysphagia are 18 times more likely to develop SaP after adjusting for age, NIHSS at admission and mRS at admission. mRS at admission is also important, while stroke severity (NIHSS) has a minor effect. Age does not significantly influence the risk of SaP once other factors are controlled (see Table 3).

**Table 3:** Multivariable logistic regression of risk factors for developing stroke-associated pneumonia.

**Variable**	**Adjusted OR (95% CI)**	***P* value**
Dysphagia	18.23 (4.02–82.54)	< 0.001
mRS at Admission	1.39 (1.04–1.85)	0.02
NIHSS at Admission	1.06 (1.00–1.12)	0.06
Age (per year)	1.02 (0.98–1.06)	0.24

Patients with dysphagia are 18 times more likely to develop SaP after adjusting for age, NIHSS at admission and mRS at admission. mRS at admission is also important, while stroke severity (NIHSS) has a minor effect. Age does not significantly influence the risk of SaP once other factors are controlled.

Patients diagnosed with dysphagia exhibited significantly poorer three-month outcomes in comparison to patients without dysphagia. A favorable outcome (mRS 0–2) was observed in 205 of 234 patients without dysphagia, but only in 45 of 93 patients with dysphagia.

Dysphagia screening and FEES use increased from 2015 to 2021 (GUSS: 14.6% → 18.0%; KSU: 48.5% → 67.1%; FEES: 1.2% → 14.7%), while dysphagia prevalence decreased (40.8% → 30.5%). Antibiotic use dropped from 22.1% in 2015 to 9.4% in 2021 (*p* < 0.001).

## 4 Discussion

CRP levels are elevated in dysphagic patients from day one, whereas in patients without dysphagia, these levels only rise slightly. The inflammatory cascade triggered by ischaemia, which releases a series of inflammatory mediators, may contribute to the slightly elevated CRP levels observed in non-dysphagic patients (Sellarès-Nadal et al., 2022). In the present study, the sensitivity of the conventional indicators of inflammation, namely the leukocyte count and the temperature, seems to be limited for the timely identification of SaP.

The recent introduction of FEES diagnostics in 2021 did not result in a significant decrease in the number of SaP cases. This is likely due to the limited number of FEES examinations conducted in 2021, which may have precluded statistical significance. However, the present study demonstrates that the establishment of FEES over time was associated with higher rates of dysphagia detection and, consequently, to more adequate therapeutic measures, coinciding with an approximate 50% decrease in antibiotic use compared to 2015.

To date, no studies have been conducted to examine the relationship between inflammation markers (e.g. CRP, white blood cells, temperature) and stroke-induced dysphagia. In mouse models, experimental induction of small ischemic brain infarcts did not result in statistically significant alterations in blood counts; however, larger infarcts induced leukopenia after 24 h, 3 days, and 7 days (Richeldi et al., 2017). The data demonstrate that there is no occurrence of leukopenia in either dysphagic or non-dysphagic patients. The leukocyte count remained within the normal range, with a slight increase observed on days 3 and 4 in patients with dysphagia. However, this increase did not reach predefined pathological levels. In non-dysphagic patients, values remained nearly unchanged, with a slight peak observed on day 4.

As demonstrated in experimental studies, there appears to be a relationship between elevated levels of C-reactive protein (CRP)—defined as greater than 15 mg/L—and a worse long-term prognosis in stroke patients (Emsley et al., 2003). In the present study, patients with dysphagia frequently exhibited a CRP level >30 mg/L at the time of admission. This finding may suggest a higher probability of early-stage infection. A high incidence of suboptimal 90-day outcomes was observed.

The early increase in CRP may be related to a minimum PAS >5 as diagnosed in FEES and the aspiration of liquids and/or food boluses in acute post-stroke dysphagia (‘material enters the airways, touches the vocal cords and is not expelled from the airways') and the aspiration of liquids and/or food boluses in acute dysphagia following a stroke. The bolus of fluid or solid food can carry chemical and infectious materials into the tracheobronchial space, hypothetically overwhelming local defenses (Liesz et al., 2009). The tissue attempts to protect itself against this aspiration-induced chemical and infectious attack through secondary macrophage activation (Abele-Horn and Pitten, 2015). Furthermore, the pharyngo-laryngeal cleansing mechanisms are insufficient and/or absent. An early experimental animal study reported the occurrence of a neutrophilic inflammatory response in pulmonary tissue within a few hours following exposure to gastric acid-containing food particles. However, the transferability of these findings to human aspiration events, particularly in the context of post-stroke dysphagia, remains unclear and warrants further investigation (VanGilder et al., 2014). There are no later studies on this subject. It is therefore not yet known as to what effect aspirated fluids and food particles have on the human lungs—a major gap in scientific knowledge.

Even healthy individuals are subject to constant microbial and particulate challenges within their respiratory ecosystem (aerosols, saliva, bodily fluids, food residues, etc.). Provided the system is functioning, the equilibrium between the attack on the pulmonary epithelium and the repair processes of the respiratory tract is sustained by elimination through coughing, throat clearing, swallowing, mucociliary clearance and the immune response. In patients suffering from a stroke, a disturbance of this balance may increase to a collapse of the system due to laryngeal dysfunction, macroaspiration and impaired clearing mechanisms (Makhnevich et al., 2019). Furthermore, hospitalization has been demonstrated to immediately elevate the risk of infection. Research conducted on nosocomial infections has indicated that pathogenic colonization of the oropharynx can occur as early as 48 h following hospitalization. Research has shown that between 30% and 40% of patients not receiving intensive care, and 70% to 75% of patients in the ICU, have Gram-negative bacteria in the oropharynx (Tuttolomondo et al., 2012).

A PAS 5 score, as diagnosed by FEES, is associated with laryngeal sensitivity and an absence of reflexive triggering of the clearing function. Clinically, the only discernible symptom of this condition is likely to be a slightly hoarse or gurgling voice. Consequently, the clinical picture is inadequate for evaluating the efficacy of the clearance of these penetrates. The screening procedures therefore only provide an indication of whether a swallowing disorder may be present and whether these patients should be referred to speech therapy for further examination. A KSU by speech therapy may provide further indications of a possible pathology of the act of swallowing. However, even in the context of speech therapy, the clinical indications are merely indicative of penetration, thus providing only a suggestion of the extent or presence of aspiration. A PAS of 1 to 2 is ‘possibly' to be suspected in the KSU, a PAS of 3 is “probably” to be suspected, but from a PAS of 4 to 5, 6, 7 and 8, only FEES provides satisfactory evidence of an insufficient clearance of possible penetrates or even aspirates.

The study's strengths include a large, clearly defined sample size and actual clinical practice. Its two different cohorts (2015 vs. 2021) allow for meaningful comparisons over time. The topic is clinically relevant and corresponds to the prevention of stroke complications. The identification of early biomarkers such as CRP in combination with the severity of dysphagia impacts patient safety and management in the stroke unit. FEES strengthens the study's practical relevance as it is the gold standard for the detection of aspiration.

However, the study also has weaknesses. Its retrospective nature limits the possibility of drawing conclusions about causality. Confounding factors may not be fully taken into account. FEES use in 2021 was too low to draw clear conclusions about FEES-related improvements in the prevention of pneumonia. There is a risk of selection bias, as dysphagia examinations were performed based on clinical suspicion. Patients with subtle or silent aspiration may have been overlooked, especially in 2015. Follow-up data (mRS after 90 days) were not available for all patients.

## 5 Conclusion

This retrospective analysis shows an association between dysphagia and elevated CRP levels during the first 4 days after stroke. Dysphagia and increased CRP were more frequently observed in patients who developed stroke-associated pneumonia (SaP), whereas leukocyte count and body temperature showed limited discriminatory value. Mild CRP elevations were also observed in non-dysphagic patients and may reflect stroke-induced immunological responses, while higher CRP levels in dysphagic patients were more often accompanied by aspiration findings on FEES. The co-occurrence of dysphagia (PAS > 5) and elevated CRP was observed more frequently in patients with SaP. This may be indicative of an early clinical constellation that warrants further diagnostic attention, rather than serving as a definitive predictive marker.

In addition, dysphagia management at the Neurology Clinic of the Münsterlingen Cantonal Hospital differed between 2015 and 2021, with increased use of FEES diagnostics in 2021. This change was accompanied by a substantial reduction in antibiotic use; Nevertheless, it is not possible to determine whether the observed association reflects improved diagnostic precision or other temporal factors with any degree of certainty within the present study.

## Data Availability

Should a legitimate interest be demonstrated, the anonymised data of the study shall be made available to the enquiring party.
